# Illinois General Hospital of the Lakes

**Published:** 1850-09

**Authors:** 


### ARTICLE IV.

### ILLINOIS GENERAL HOSPITAL OF THE LAKES.

This institution, which was chartered by the recent extra-session of the Illinois Legislature will go into full operation on the first of October next. It would have been opened much earlier, but for the difficulty of securing an appropriate building. This important object has now been accomplished, and the spacious edifice known as Tippecanoe Hall, situated in a central part of the city has been secured for its use. The most liberal arrangements will be made for the accommodation of patients, so that persons coming from a distance to submit to treatment may be assured of finding accommodations and care on moderate terms.

By the law incorporating the Hospital, the County Boards of the different Counties in the State are authorized to send cases to the Hospital, provided the charge shall be at the lowest possible price consistent with the care they receive.

The best medical attention will be secured to patients in the Hospital, free of charge.

The provisions of the charter are of the most liberal character, and the Board of Trustees are determined to carry out to the fullest extent the benevolent designs of the Legislature.

We understand a circular will shortly be issued by the Trustees, giving a full detail of its organization, together with the terms of admission, &c.

Thus has been commenced an institution which, we trust, with the smiles of an over-ruling Providence, is destined to a long career of usefulness in relieving sickness, pain and sorrow, and which will be a monument to the memory of those who contribute to its endowment and support, more honorable than the laurels of the conquerer. E.

